# Increased Urgent Care Center Visits by Southeast European Migrants: A Retrospective, Controlled Trial from Switzerland

**DOI:** 10.3390/ijerph15091857

**Published:** 2018-08-28

**Authors:** Jolanta Klukowska-Röetzler, Maria Eracleous, Martin Müller, David S. Srivastava, Gert Krummrey, Osnat Keidar, Aristomenis K. Exadaktylos

**Affiliations:** 1 Department of Emergency Medicine, University Hospital, 3010 Berne, Switzerland; martin.mueller@extern.insel.ch (M.M.); DavidShiva.Srivastava@insel.ch (D.S.S.); Gert.Krummrey@insel.ch (G.K.); osnatjacob@gmail.com (O.K.); Aristomenis.Exadaktylos@insel.ch (A.K.E.); 2 Department of Rheumatology, Immunology and Allergology, University Hospital, 3010 Bern, Switzerland; maria.eracleous@insel.ch; 3 Institute of Health Economics and Clinical Epidemiology, University Hospital, 50935 Cologne, Germany

**Keywords:** Southeast Europe, immigrant, healthcare

## Abstract

We investigated whether immigrants from Southeast Europe (SE) and Swiss patients have different reasons for visiting the emergency department (ED). Our retrospective data analysis for the years 2013–2017 describes the pattern of ED consultations for immigrants from SE living in Switzerland (Canton Bern), in comparison with Swiss nationals, with a focus on type of referral and reason for admission. A total of 153,320 Swiss citizens and 12,852 immigrants from SE were included in the study. The mean age was 51.30 (SD = 21.13) years for the Swiss patients and 39.70 (SD = 15.87) years for the SE patients. For some countries of origin (Albania, Bosnia and Herzegovina, and Turkey), there were highly statistically significant differences in sex distribution, with a predominance of males. SE immigrants had a greater proportion of patients in the lower triage level (level 3: SE: 67.3% vs. Swiss: 56.0%) and a greater proportion of patients in the high triage level than the Swiss population (level 1: SE: 3.4% vs. Swiss: 8.8%). SE patients of working age (16–65 years) were six times more often admitted by ambulance than older (≥65 years) SE patients, whereas this ratio was similar in the Swiss population. In both groups, the fast track service was primarily used for patients of working age (<65) and more than three times more often in the SE than the Swiss group (SE: 39.1%, Swiss: 12.6%). We identified some indications for access to primary care in emergency departments for immigrants and highlighted the need for attention to the role of organizational characteristics of primary health care in Switzerland. We highlighted the need for professional support to improve the quality of healthcare for immigrants. In the future, we will need more primary care services and general practitioners with a migrant background.

## 1. Introduction

Switzerland is among the countries in Europe with the highest percentage of foreigners in its permanent population [1]. According to the information of the Swiss Federal Statistical Office (OFS) for the end of 2017, the Swiss population of 8,482,200 citizens included a high proportion of immigrants (2,108,001; 24.8%) [2]. The most common European country of origin was Italy (19.0%), followed by Serbia, Montenegro, and Kosovo (each 13.0%), Portugal (11.0%), and Germany (10.0%). Current immigration policy in Switzerland favors qualified workers from the European Union (EU) and particularly from Southeast Europe (SE) [3]. Most migrants from SE come from Kosovo (5.3%; *n* = 112,233), Turkey (3.2%: *n* = 67,460), Macedonia (3.1%; *n* = 65,893), and Serbia (3.0%; *n* = 63,493) (Table 1).

Migration health is a specialized field of health sciences and focuses on the well-being of migrants. Migrants in a state of well-being are more receptive to education and employment [4]. They are not perceived to be a health threat to host societies, are less exposed to discrimination and are more likely to be accepted as equal citizens [4]. In various recent conventions and declarations (UN, WHO, EU), countries (including Switzerland) are called upon to work towards equality of opportunity in health. In order to improve the health status of the migrant population in Switzerland, the Confederation launched the “Migration and Public Health Strategy, 2008–2013”, under the auspices of the FOPH. Various federal offices and federal agencies, as well as other organizations (Committee on the Elimination of Racial Discrimination, World Health Organization), have been involved in implementing the following strategy: “Everyone living in Switzerland shall be given a fair opportunity to develop their health potential. No-one will be disadvantaged by avoidable discrimination” [5].

In research on migration and health, questions about the health status and health-related behavior of the migrant population—and their causes and effects—are studied. However, there are gaps in current knowledge in this research area. A few publications have explored differences between immigrants and nationals [6,7,8,9,10,11], socioeconomic status [6], or general well-being and health of short- and long-term immigrants [7,12,13,14]. Some studies have found that immigrants attend EDs more often than host populations—but these findings have not been consistent [15,16]. Several studies have analyzed the health of immigrant patients in Switzerland and have reported specific health-related problems [6,8,9,17,18,19,20]. 

The first part of this study was aimed at describing the characteristics of SE immigrant patients admitted to our ED, in comparison to Swiss patients. The second part compared types of referral, reasons for admission, and triage of Swiss and SE patients.

## 2. Material and Methods

### 2.1. Setting

This study covers the city and canton of Bern, in central Switzerland. The study site is a level 1 interdisciplinary university ED, caring for more than 2 million people and treating about 42,000 patients (2017) of all social classes and insurance groups per year.

### 2.2. Study Design

This is a retrospective cohort study based on the demographic and health data of the patients admitted to the ED in Bern University Hospital from 1 January 2013 to 31 December 2017. Patients younger than 16 years are generally treated in the pediatric clinic and were therefore not included in this study.

### 2.3. Data Collection and Extraction

All data were extracted from the routine records of the digital data base system E.care (E.care BVBA, ED 2.1.3.0, Turnhout, Belgium). All patients were grouped according to nationality. Patients were classified into two groups: immigrants from SE without Swiss citizenship and Swiss citizens. Patients with other nationalities were excluded from this study. In these studies, we could not differentiate between native Swiss citizens and naturalized foreigners.

In accordance with the classification of SE by the European Travel Commission and the Danube-Sava definition [21,22], the SE immigrant group included all patients from: Albania, Bosnia and Herzegovina, Bulgaria, Croatia, Greece, Hungary, Kosovo, Macedonia, Moldova, Montenegro, Romania, Serbia, Slovenia, and Turkey. Although it is largely in Asia, we included Turkey in this study under SE. Relations between the European Union (EU) and Turkey were established in 1959 and Turkey is one of the EU’s main partners in the southeast, and since 1987 Turkey has been an applicant to accede to EU. 

Furthermore, demographic data (age, gender) and clinical data, including admission data, triage, reason for admission, and type of referral, were extracted from the ED’s electronic database for patients. 

The patients were classified into the following age classes: 16–25, 25–35, 35–45, 45–55, 55–65, 65–75, 75–85, 85–95, 95–105; genders (male, female); triage (1, 2, 3, 4, 5); type of referral (ambulance, air rescue, general practitioner, external hospital, walk-in, repatriation, military, police, and internal referral); and reason for admission (surgery, internal medicine, fast track, psychiatry). Fast track services are designed for patients seeking primary care services for less serious illnesses and injuries.

Patients in our ED are routinely triaged using an abbreviated version of the Manchester Triage System [23]. This triage system classifies the urgency of treatment for patients presenting to an ED in five levels: 1: acute life threating problem (immediate treatment required), 2: high urgency, 3: urgency, 4: less urgency, 5: no urgency. When a new patient presents to the ED, a specially trained nurse assigns the patient’s reported complaints according to a defined algorithm and then determines the treatment priority with the aid of fixed rules that take into account the vital signs.

### 2.4. Definitions

In this study, ‘immigrant’ is defined as any foreign person according to the Swiss law on citizenship. A first-generation immigrant is someone who has moved to Switzerland after being born elsewhere. A second-generation immigrant is someone born to first-generation immigrants. Swiss citizenship is the status of being a citizen of Switzerland and can be obtained by birth or naturalization. People who are not born or naturalized in Switzerland were classified by their country of origin. The citizenship status is routinely assessed by our hospital administration system.

### 2.5. Ethical Considerations

This descriptive retrospective study was approved by the cantonal (district) ethics committee in Berne, (No. 2018-00198). No individual informed consent was obtained. The analysis was carried out with anonymized data.

### 2.6. Statistical Analysis

All data were presented as frequencies and percentages. The data were summarized using descriptive statistics. Data analysis was performed using Stata 13.1 (StataCorp, The College Station, TX, USA). Differences between patient groups were tested using the chi-square test. 

## 3. Results

### 3.1. Demographic Distribution

**Figure 1:**
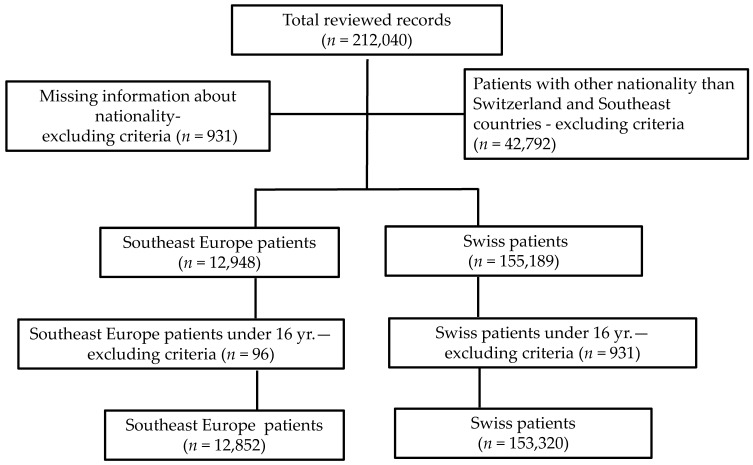
Flow chart of medical record selection.

A total of 12,852 immigrants from SE were admitted to the ED during the five-year study period. Over the same period, 153,320 Swiss citizens used our ED services (Table 2). Patients of other nationalities were excluded from this study (42,972). Some consultations were excluded from the analysis because key demographic information (nationality) was omitted in the patient information system (*n* = 931) or the patients were younger than 16 years old (SE immigrant patients: *n* = 96, Swiss: *n* = 1869). Thus, the total number of consultations included in the analysis was 166,172 (Figure 1).

**Figure 2:**
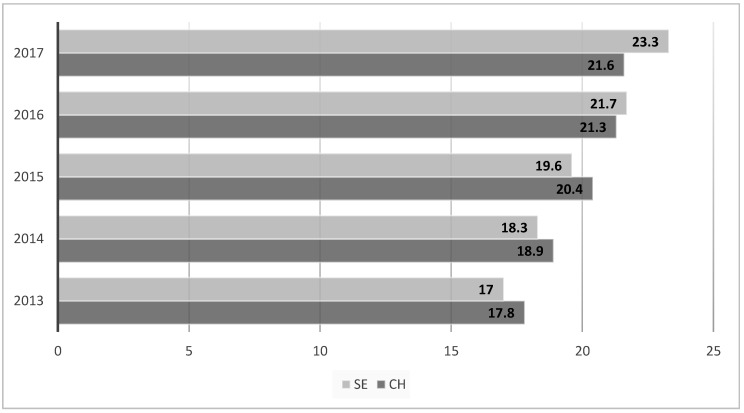
Percentage annual distribution of patients between 2013 and 2017 (SE: Southeast Europe; CH: Switzerland).

An increase in the annual number of patients was recorded in both analyzed groups over the study period: Swiss *n* = 33,074 and SE *n* = 3021 in 2017 compared to 27,218 (Swiss) and 2208 (SE) in 2013 (Figure 2). The largest group of SE patients were from Turkey (*n* = 2870, 22.3%), followed by Macedonia (*n* = 2078, 16.2%), and Kosovo (*n* = 2233, 15.8%) (Table 2).

### 3.2. Gender Distribution

More than half of the Swiss patients were men (55.6%) and 44.4% were women. Similarly, 53.9% of the SE patients were men and 46.1% women. For most SE countries, most patients were male (max. Moldava: 70.0%). However, patients from some countries included many women (Bulgaria: 59.4%, Hungary 57.0%) (Table 2). There were highly significant differences (*p* < 0.0001) from the Swiss population in patients from Albania, Bosnia and Herzegovina, and Turkey and significant differences (*p* < 0.05) in patients from Bulgaria, Greece, Hungary, and Serbia. 

### 3.3. Age Distribution

**Figure 3:**
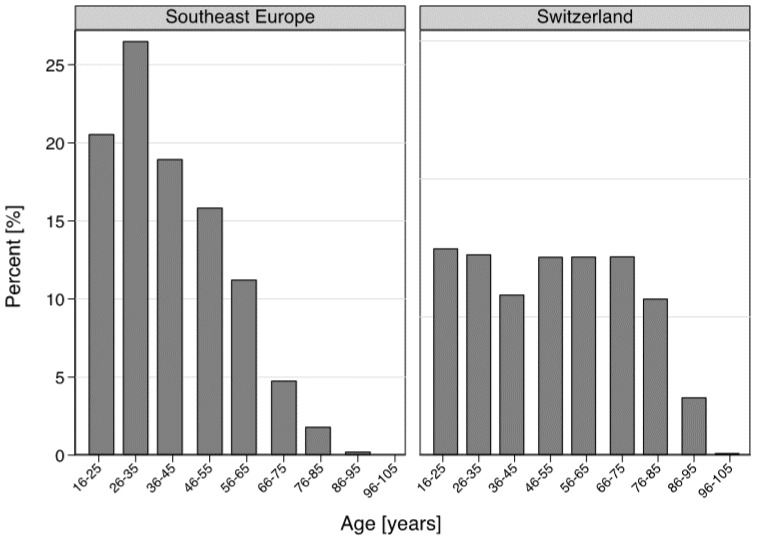
Comparison of the age distribution between Swiss and Southeast Europe patients.

The mean age of the Swiss population was 51.30 (SD = 21.13), in comparison with 39.70 (SD = 15.87) in the SE population (*p* < 0.0001). Figure 3 highlights the age data of the cohort of ED patients studied. Between 2013 and 2017, 30.1% of the Swiss patients were older than 66 years, compared to 6.8% of the SE population (6.8%). Young adults of working age (16–65) were more common in the SE group (93.2% vs. 69.9% in the Swiss group). Very old patients (≥85) were represented only in the Swiss population (4.3%, *n* = 6655, including 26 centenarians, in contrast to only 29 patients (0.2%) in the SE group, including only six patients older than 90).

### 3.4. Triage

**Figure 4:**
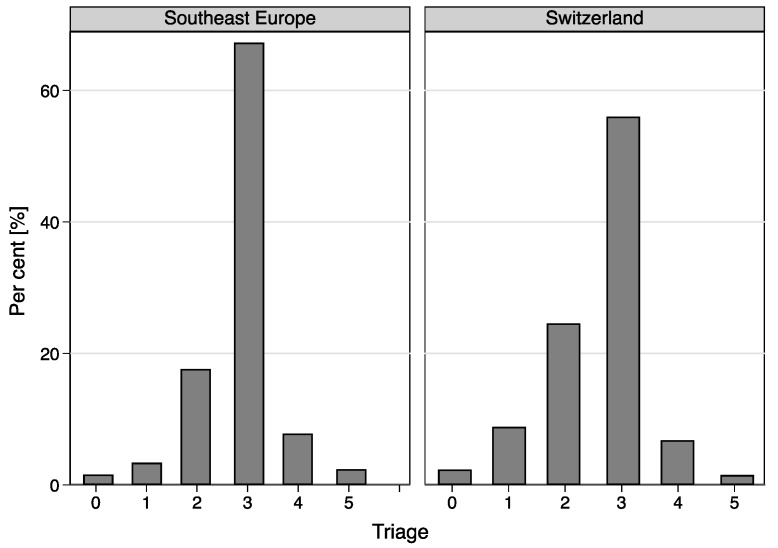
Comparison of triage distribution between Swiss and Southeast Europe immigrant groups.

The SE immigrant group exhibited different levels of triage, with a higher proportion in the lower triage level 3 (level 3: 67.3% vs. 56.0% in the Swiss group). Correspondingly, there were more patients in the high triage level in the Swiss group (level 1: Swiss: 8.8%, SE: 3.4%; level 2: Swiss 24.6%, SE: 17.6%) (Figure 4). There was a significant association between the triage level and immigration from SE (*p* < 0.0001). The mean triage level in patients from SE was 2.84 (95% CI: 2.82–2.85), but from Switzerland 2.61 (95% CI: 2.60–2.61) (*p* < 0.001).

Following the ED consultation, 65.5% of Swiss patients were treated as outpatients and 34.5% were hospitalized. SE immigrant patients were hospitalized less often (21.0%). There was a general trend that higher triage levels (1, 2) were associated with higher hospitalization rates (Swiss population: 48.9% vs. SE: 38.0%).

### 3.5. Type of Referral

**Figure 5:**
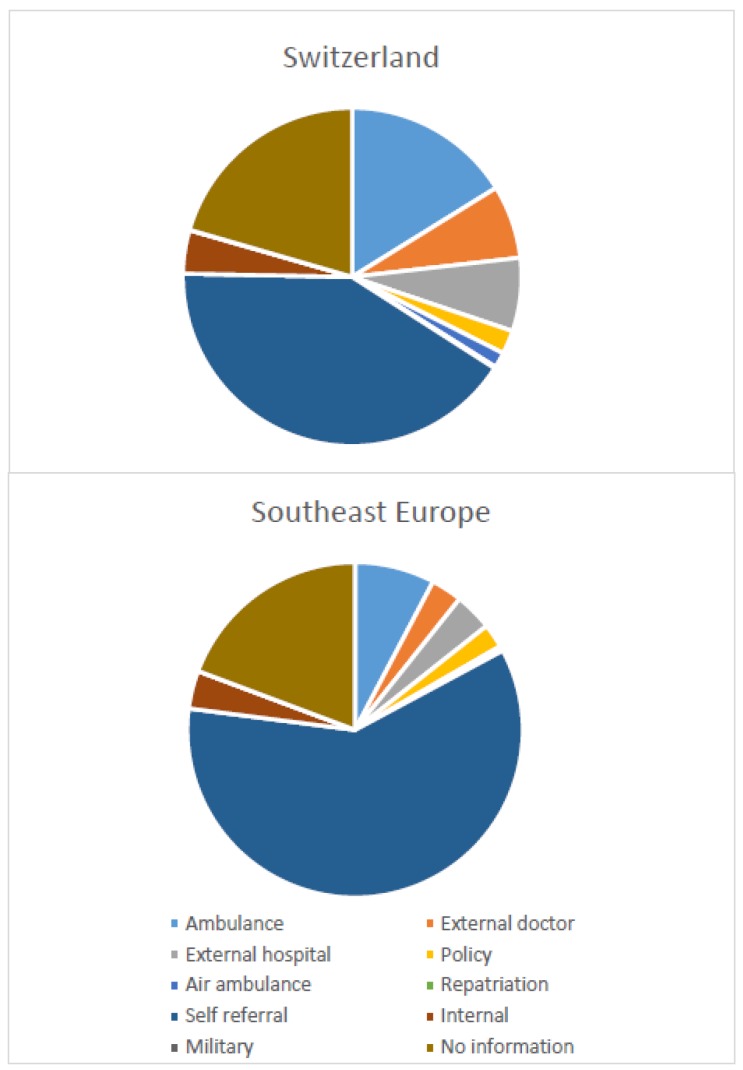
Comparison of type of referral between Swiss and Southeast Europe immigrant populations.

The main difference in the type of referral was observed in the self-referral group, which was more frequent with the SE immigrants (59.9%) than in the Swiss patient population (41.2%) (Figure 5). In contrast, referral by ambulance was more frequent in the Swiss patients than in the SE group (16.2% vs. 7.7%). SE patients of working age (16–65 years) were six times more often admitted by ambulance than were old (≥66) SE patients (86.8% vs. 13.2%), whereas in the Swiss population this ratio was similar. Swiss patients were transferred twice more often from an external hospital or an external doctor than SE patients (external hospital, Swiss 7.0% vs. SE 3.6%; external doctor Swiss 7.0% vs. SE 3.0%).

### 3.6. Reason for Admission

**Figure 6:**
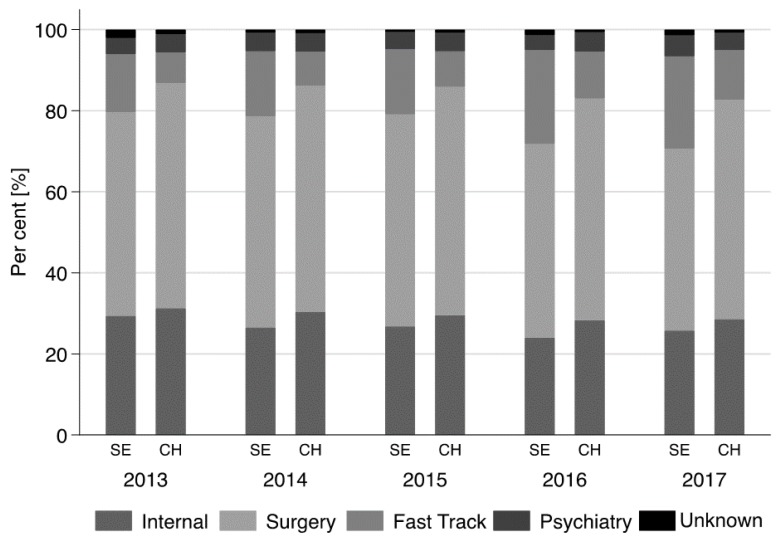
Reason for admission between 2013–2107 (SE: Southeast Europe; CH: Switzerland).

A highly significant association was found between ‘reason for admission’ and immigration from SE (*p* < 0.001). About 55.0% of Swiss patients (55.3%, *n* = 84,717) presented with internal medical complaints, 29.5% (*n* = 45,284) with surgical complaints, and 4.5% with psychiatric complaints. Almost 10.0% of Swiss patients (9.9%, *n* = 15,127) used a medical service in the fast track section of ED. These values differed significantly in the SE immigrant group: medical: 48.2% (*n* = 6326), surgery: 26.4% (*n* = 3388), psychiatry: 4.4% (*n* = 559), and fast track: 18.9% (*n* = 2423) (Figure 6).

In both groups, admission for internal medicine and surgical complaints increased steadily by about 3.0% during the five-year period. Within the same period, the total number of admitted patients increased from 38,027 in 2013 to 46,059 in 2017.

In the Swiss population, admission for internal medical and psychiatric complaints were predominant in Swiss patients between 16 and 65 years (working age population). In both study populations, the fast track service was used primarily by patients of 65 years and younger, corresponding to 12.6% of the Swiss population and 39.1% of SE immigrants. In patients aged >65, the use fast track was as follows: Swiss: 3.5% and SE immigrants 8.1%.

## 4. Discussion

### 4.1. Population of SE Immigrant Background in Switzerland

SE is a relatively new geopolitical denotation for the Balkan states, a region frequently regarded by Western countries as a heterogeneous set of countries with their own cultural specific features, dynamics, and an interconnected and complex modern history. There are many overlapping and conflicting definitions as to where exactly SE begins or ends or how it relates to other regions of the continent. The countries that form this part of Europe are Albania, Bosnia and Herzegovina, Bulgaria, Croatia, Kosovo, Macedonia, Moldova, Montenegro, Romania, Serbia, Slovenia, and—to some extent—Greece, Hungary, and Turkey [21].

After the Second World War, many refugees from countries involved in wars, including people from SE, found asylum in Switzerland. On the other hand, throughout the 20th century, immigration to Switzerland was organized around the needs of the domestic labor market and counteracted the shortage of (mostly unskilled) labor by hiring foreign workers. 

For example, an anti-Communist revolt in Hungary started in 1956. Hundreds of thousands of people left the country, with around 14,000 seeking safety in Switzerland. The refugees were initially accommodated in army barracks, public buildings, hotels, and guesthouses. Over the following months, they were dispersed over all the Swiss cantons. Roughly 23,313 Hungarian citizens are now resident in Switzerland (2017) [2]. Most of these are former Hungarian refugees who chose to return to their country of origin after reaching retirement age in Switzerland. In comparison, Greece and Switzerland have a long relationship. During the rule of the military junta in Greece (1967–1974), many members of the opposition found protection in Switzerland. About 13,000 Greeks now live in Switzerland (2017); in 2010, there were 6808 [2]. Greeks who live permanently in Switzerland work in a variety of professions, such as medicine, banks, universities, organizations in the EU and the UN, where they excel and hold prominent and senior positions. The most numerous group of Southeast European immigrants (about 300,000 in 2017) arrived from the former Yugoslavia to live in Switzerland [2]. About half of these are Albanians (mostly Kosovar Albanians and to a lesser extent Albanians from Macedonia); the other half is made up of South Slavic groups (Serbs and Bosnians) and lower numbers of Croats, Macedonians, and Slovenes. Large numbers of workers obtained long-term permits between 1985 and 1998 and the subsequent inflow of family members generated the largest increase in the Yugoslav population in Switzerland.

Since immigration of skilled workers was initially promoted in the 1990s, and—as a result of the bilateral agreements with the EU—the proportion of well-educated and higher earning migrants has been increasing. At the same time, there are still high rates of immigration in industries with low levels of qualification (agriculture). Male and female foreign workers are very strongly represented in the construction sector and the hotel and restaurant industry, as well as in the healthcare sector. Foreign women are found in the sex industry and still often work illegally as domestics. Differences between Swiss citizens and SE immigrants are reflected in income. On average, the worst paid individuals in Switzerland are workers originating from the former Yugoslavia. The difference results from the individual level of education. Half of these immigrant workers from the Balkans did not receive any further training after completing obligatory school education. Unfortunately, that is also reflected in their quality of life and social status. Thirty percent of immigrants from the Balkans, Turkey, Romania, and Bulgaria are affected by poverty, whereas just under 20% from SE and only 7% from northern and western Europe are in poverty [5].

Many of the immigrants who arrived some time ago have stayed in Switzerland with their families, and these children born in Switzerland belong are now second-generation immigrants. On the one hand, there is an accumulation of risks and problems relating to the health; on the other hand, the second generation lives almost identically to the native population, with much better education and health conditions.

Immigrants may be very heterogeneous in some host countries and that the conventional Western conception of immigrants as vulnerable individuals characterized by low socio-economic status—working in unhealthy jobs, having poor health literacy and poor access to health services—may not necessarily be generalized [24]. Established theoretical explanations on migration and health can account for such differences: social and cultural patterns from the country of origin shape physical activity, body images, dietary intake, and food preferences [25,26]. Volhen and Rüesch reported that in the Switzlerland poor health and activities of daily living impairments were consistently associated with low socioeconomic status, low sense of mastery and little social support. Immigrant-specific preventive and health promotion initiatives should therefore target these immigrant groups [24]. 

As a result of their poorer overall socio-economic status compared with the native Swiss population, the migrant population has twice the rate of poverty (21.4%) and is also over-represented in the group of the working poor. A low level of education, unfavorable working conditions, or unemployment expose female foreigners to a particularly high risk of poverty. However, there are enormous differences in the poverty statistics depending on the country of origin. For instance, 30% of immigrants from the western Balkans—from Turkey, Romania, and Bulgaria—are affected by poverty, whereas just under 20% from southern Europe and only 7% from northern and western Europe are in poverty. It is hardly surprising that foreigners resident in Switzerland claim state benefits far more often than native Swiss men and women [5].

### 4.2. Health Care of SE Immigrants

In Switzerland, the foreign population is relatively young: for every 100 foreigners of working age (aged 20–65) there are only 11 aged 66 and over (compared with 36 among the Swiss) [27]. In our study, the mean age of SE immigrant patients was 39.7 (SD = 15.87) years, in contrast to the value for the Swiss group: 51.3 (SD = 21.13) years. The migrant groups from Turkey and former Yugoslavia contain a particularly high proportion of young people. Between 2013 and 2017, 1371 people from the 14 SE countries were awarded Swiss citizenship, mostly young people between 16–45 years (1207 applications) [2]. In the same time period, 1812 people decided for re-emigration. Some authors reported that people using the ED as their primary source of health care were significantly younger, which corresponds to our results [28]. Older patients more often favor the general practitioner for referral to the ED, rather than using the outpatient clinic [29]. This is consistent with our finding that younger patients of occupational age tend to visit the fast track. 

Our data indicate that immigrants from SE to Switzerland—including both first- and second-generation immigrants—use the walk-in emergency services more often than Swiss patients. They tended to use the fast track (primary care) services in our emergency clinic, whereas the proportion of SE immigrants at the trauma clinic (internal medicine, surgery) was similar to the group’s representation in the Swiss patient population. Between 2013 and 2017, the annual number of psychiatric emergencies among immigrants has tended to increase (by 70.0% in the five-year period). In the same period, the number of psychiatric cases in the native population increased by 17.8%. Similar findings have been seen in other studies on psychiatric problems among immigrants in ED [9,30]. As a comparison, other Swiss studies have found increasing numbers of psychiatric patients in ED (from 60 to 315 per year between 2007 and 2012) and the most common reasons for psychiatric presentation to ED were psychosis (20.3%), social problems (18.2%), auto-aggression (16.4%), and depression (16.2%) [9]. Cultural factors can play an important role in the psychological response to stress. The process of integration is certainly associated with stress and higher vulnerability for mental health problems [31,32]. Risk factors for mental health in the migration group are associated with living condition in the country of origin, duration of the migration, living condition in the country of immigration, sometimes with feelings of intolerance, the legal or social frameworks, and communications problems. Low social status (low incomes) are often associated with health-related behaviors like drinking and smoking and these may put physical and mental health at risk [31].

Previous publications on healthcare and migrants in Europe and Switzerland showed that migrants tend to have more contacts with general practitioners than the original population does [33,34]. In the present study, SE migrants more often carried out walk-in medical visits in ED than did the Swiss population. Walk-in SE patients more often use services in the fast track division (22.8% versus 15.8% in the Swiss population). Fast track services are designed for patients seeking primary care services (often without a general practitioner) for less serious illnesses and injuries. International studies have suggested that immigrants use emergency services more for non-urgent health care problems than do native populations [16,35,36]. A Norwegian study reported that a frequent reason for not contacting a general practitioner before the emergency department was because it was difficult to access him. In this study, 21% of the native Norwegians and 4% of the immigrants stated they had a general practitioner in another district and 33% of immigrants did not have their own general practitioner. In the same study, a high percentage of immigrants from Turkey (41.0%) and Africa (41.0%) had a problem in contacting any general practitioner [35]. Immigrant patients have frequently different reasons for underutilizing health services or the local health system, including poor education and lack of language skills. These impediments may lead to the wrong access to health care, so that immigrants may utilize ED services for non-urgent cases that can be treated in primary care settings [15]. This is consistent with our finding that the distribution of triage depends on the study population. During the analyzed period, immigrants were more likely to be frequent fast track users compared with Swiss population, although there were differences between immigrant groups. Older immigrants used fast track less often, whereas immigrants in the worked age were over-represented among frequent attenders.

The foreign population is on average younger than the Swiss population. Switzerland’s population continues to age. 18.1% of the population is over 64, while there are 29 people over 64 for every 100 working-age people—those between the ages of 20 and 64 [34]. Taken as a whole, this may help to explain why most immigrants >65 years used their general practitioners less than younger immigrants and Swiss patients. As explained above, our study identifies the oldest immigrants as small group of patients in ED compared with numerous younger immigrants and Swiss patients. Although this could be partially explained by remigration to the country of origin after retirement. Our results are in contrast with other studies, where older immigrants used more health services than natives [35]. Thus, this group should be further studied, as a group with more access barriers to ED. This group will probably increase in the future as second and subsequent generations of SE immigrants adapt to living conditions in Switzerland and are not motivated to return to their country of origin.

### 4.3. Limitations

Our study has some limitations. We decided to include all generation SE immigrants as one group in our study. As a result, we might have missed important differences between the first and second generations, who are generally better integrated and have a more similar lifestyle to the native population than did their parents. According information from the Federal Statistical Office FSO Section Population in District Bern for 2012–2016, ca. 96% of SE immigrants were first generation and only 7% second generation.

We only included data from an ED in central Switzerland, where the annual number of patients, including SE immigrants, is higher than in other emergency centers in private hospitals in Bern, or in the rest of the country. This might influence the choice of ED in case of an emergency. Other nationalities (except SE and Swiss) are excluded from the study, because Switzerland has a high percentage of immigrants and our goal was to characterize a SE patient population. 

We restricted our analysis to adult patients. Thus, children (<16 years) were not included in the analysis. Furthermore, women with pregnancy- and delivery-related complications were not included in this study, as they were admitted directly to the Department of Obstetrics and Gynecology.

## 5. Conclusions

Between 2013 and 2007, SE immigrants used ED services differently than did Swiss citizens, depending on their nation of origin. Immigrants more often use the ED for low urgency complaints and this may suggest that barriers to primary healthcare may be driving the greater use of these services.

In Bern, immigrant subgroups use emergency services differently. Increased use was seen mostly at the fast track clinic, whereas the proportion of immigrants at the trauma and internal medicine was similar to the Swiss population. Immigrants of working age from SE used the fast track in the emergency department more frequently than the Swiss did. These different patterns of health-seeking behavior are important when planning and designing emergency and primary health care services for immigrants in large cities such as Bern.

We identified some differences in access to primary care in an emergency department for immigrants and emphasized the need for attention to the role of organizational characteristics of primary health care in the Switzerland. We highlighted the need for professional support to improve the quality of healthcare for immigrants. In the future, more primary care services and general practitioners with a migrant background must be provided. 

Swiss primary health care needs to evolve to address the challenges of migrant populations. We hope that our results provide indications for practices and health systems interested in improving health care delivery for this vulnerable population. As numerous migrants with low income move to smaller cities, the primary health system must find ways to implement interpretation services, support comprehensive care and continuity of care, provide guidelines, and develop training for practitioners. On the other hand, in the future access to preventive care will be particularly important for immigrant patients, as this is a determinant of the future risk of chronic disease, which in turn may lead to socioeconomic disadvantage.

## Figures and Tables

**Table 1:** Number of Southeast Europe citizens (SE) in Switzerland and in Canton Bern and their percentage in comparison to the total number of immigrants [2].

Country	Switzerland	%	Canton Bern	%
Total number of immigrants	2,108,001		159,617	
Albania	1824	0.1	176	0.1
Bosnia and Herzegovina	30,282	1.4	1919	1.2
Bulgaria	9869	0.5	1145	0.7
Croatia	29,081	1.4	2419	1.5
Greece	13,684	0.7	616	0.4
Hungary	23,313	1.1	1846	1.2
Kosovo	112,233	5.3	8674	5.4
Macedonia	65,893	3.1	6378	4.0
Moldova	611	0.0	51	0.0
Montenegro	2517	0.1	0	0.0
Romania	18,092	0.9	1633	1.0
Serbia	63,493	3.0	4071	2.6
Slovenia	6753	0.3	408	0.3
Turkey	67,460	3.2	5392	3.4

**Table 2:** Comparison of the gender distribution in the Swiss and Southeast Europe groups and in the individual SE group countries.

Country of Origin	Male		Female		Total	
	*n*	%	*n*	%	*n*	%
**Switzerland**	85,195	55.6	68,125	44.4	153,320	100
**Southeast Europe**	6928	53.9	5924	46.1	12,852	100
Albania	672	61.9	414	38.1	1086	8.5
Bosnia and Herzegovina	455	59.4	311	40.6	766	6.0
Bulgaria	123	40.6	180	59.4	303	2.4
Croatia	379	51.8	353	48.2	732	5.7
Greece	115	58.7	81	41.3	196	1.5
Hungary	144	43	191	57.0	335	2.6
Kosovo	1011	49.7	1022	50.3	2033	15.8
Macedonia	1083	52.1	995	47.9	2078	16.2
Moldova	14	70.0	6	30.0	20	0.2
Montenegro	23	50.0	23	50.0	46	0.4
Romania	230	49.9	231	50.1	461	3.6
Serbia	959	52.7	861	47.3	1820	14.2
Slovenia	57	53.8	49	46.2	106	0.8
Turkey	1663	57.9	1207	42.1	2870	22.3
